# Isolation and characterization of patient-derived CNS metastasis-associated stromal cell lines

**DOI:** 10.1038/s41388-019-0680-2

**Published:** 2019-01-30

**Authors:** Ben Yi Tew, Christophe Legendre, Gerald C. Gooden, Kyle N. Johnson, Rae Anne Martinez, Jeff Kiefer, Mark Bernstein, Jennifer Glen, Loren Butry, Aleksander Hinek, Steven A. Toms, Bodour Salhia

**Affiliations:** 1 Department of Translational Genomics, Keck School of Medicine, Norris Comprehensive Cancer Center, University of Southern California, Los Angeles, CA USA; 2 Translational Genomics Research Institute, Phoenix, AZ USA; 3 Division of Neurosurgery, University Health Network, Toronto, ON Canada; 4 Peter Gilgan Centre for Research and Learning, Hospital for Sickkids, Toronto, ON Canada; 5 Department of Neurosurgery, Geisinger Health System, Danville, PA USA; 6 Department of Neurosurgery, Lifespan Health System and Warren Alpert School of Medicine, Brown University, Providence, USA

**Keywords:** Cancer genomics, CNS cancer, Breast cancer, Cancer models, Cancer microenvironment

## Abstract

The functional role of human derived stromal cells in the tumor microenviornment of CNS metastases (CM) remain understudied. The purpose of the current study was to isolate and characterize stromal cells of the tumor microenvironment in CM. Four different patient-derived cell lines (PDCs) of stromal and one PDC of tumorigenic origin were generated from breast or lung CM. PDCs were analyzed by DNA/RNA sequencing, DNA methylation profiling, and immunophenotypic assays. The stromal derived PDCs were termed CNS metastasis-associated stromal cells (cMASCs). Functional analysis of cMASCs was tested by co-implanting them with tumorigenic cells in mice. cMASCs displayed normal genotypes compared with tumorigenic cell lines. RNA-seq and DNA methylation analyses demonstrated that cMASCs highly resembled each other, suggesting a common cell of origin. Additionally, cMASCs revealed gene expression signatures associated with cancer associated fibroblasts (CAFs), epithelial to mesenchymal transition, mesenchymal stem cells and expressed high levels of collagen. Functionally, cMASCs restricted tumor growth, and induced desmoplasia in vivo, suggesting that cMASCs may promote a protective host response to impede tumor growth. In summary, we demonstrated the isolation, molecular characterization and functional role of human derived cMASCs, a subpopulation of cells in the microenvironment of CM that have tumor inhibitory functions.

## Introduction

Central nervous system metastases (CM) are the most common group of intracranial tumors, occurring in 15–40% of all cancer patients with metastatic disease [1–3]. Lung and breast cancer are the most common primary tumors that metastasize to the central nervous system (CNS) [1–3]. The rising incidence of CM in recent years is likely due to prolonged survival of cancer patients receiving aggressive treatments for their primary or systemic disease [1–3]. Proven therapies for CM are restricted to palliative radiation and surgical resection. Traditional chemotherapy, targeted inhibitors, and immunotherapy remain unproven for CM patients and there are few options for clinical trials. Survival benefit from these options is limited, and the two-year survival rate remains dismally below 2% [4, 5].

The progression of primary tumors towards metastasis is a multistage process in which malignant cells spread and colonize a distant organ in a series of sequential steps described as the metastatic cascade [6]. Specifically, in CM, tumor cells embolize to distant vessels and invade across the blood-brain barrier arriving in a dynamic cellular and molecular landscape that presents unique selection pressures. Metastatic tumor cells have to adapt to this vastly different microenvironment in the brain, including different immune cell and extracellular matrix composition in the brain parenchyma [7]. An extensive body of clinical data and experimental research has reaffirmed the original “seed and soil” hypothesis proposing that the organ-preference patterns of tumor metastasis are the product of favorable interactions between metastatic tumor cells (the “seed”) and their organ microenvironment (the “soil”) [8, 9]. After gaining access to the soil of the new tissue, metastatic cells interact with resident cells in the tissue and immune cells to promote the growth of the new metastasis [10]. For example, synthesis of proangiogenic proteins can promote adjacent microvascular endothelial cells to form new vascular networks or recruit bone marrow derived endothelial precursors [11]. Also, several recent studies suggest that some stromal cells within metastases may actually participate in a process resembling a wound healing response [12, 13], whereby changes to the brain microenvironment has been shown to inhibit tumor growth [14, 15].

Still, there remains a paucity of research on the interaction of the tumor microenvironment with disseminated tumor cells in CM. Studies have been limited to immunohistochemical analyses or in vivo animal models that do not explicate the complexity of the human disease. Therefore, in the current study we report on the isolation, molecular and functional characterization of stromal cells of the CM tumor microenvironment.

## Results

### Isolation and molecular characterization of novel non-tumor and tumor patient-derived cell lines from lung and breast cancer CNS metastases

In an attempt to generate new cell lines from patients with breast or lung CM, cells were isolated and cultured from a series of surgically resected CM tissue samples. Five different patient-derived cell lines (PDCs) from patients with lung or breast cancer CM were established: two lung adenocarcinomas to brain (CM03, CM08), one lung adenocarcinoma to spine (CM02), one small cell lung carcinoma (SCLC) to brain (CM04), and one breast carcinoma to brain (CM01). Additionally, a patient-derived xenograft cell line (PDC-X) was established from CM01. Patient demographic data is presented in Suppl Table 1. To ascertain the cellular lineage of the five CM PDCs and one PDC-X, we performed exome-seq, RNA-seq, and DNA methylation analysis.

**Fig. 1:**
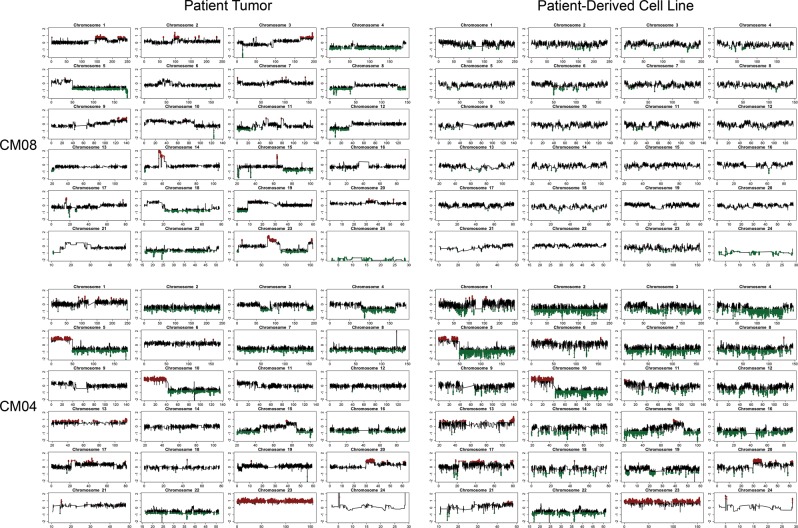
Copy number aberration profiles for non-tumoringenic (CM08) and tumorigenic (CM04) PDCs (right) and their patient matched-tumors (left)

Copy number analysis of CM01, CM02, CM03, and CM08-PDCs revealed profiles with minimal genomic aberrations resembling a normal cell profile (Fig. 1, Suppl Fig. 1). CM04-PDC highly resembled its patient tumor, which showed corresponding gain of the p-arm of chromosome 5, loss of the q-arm of chromosome 10 (including a homozygous deletion of PTEN), among other copy number changes consistent with the original patient tumor (Fig. 1).

**Fig. 2:**
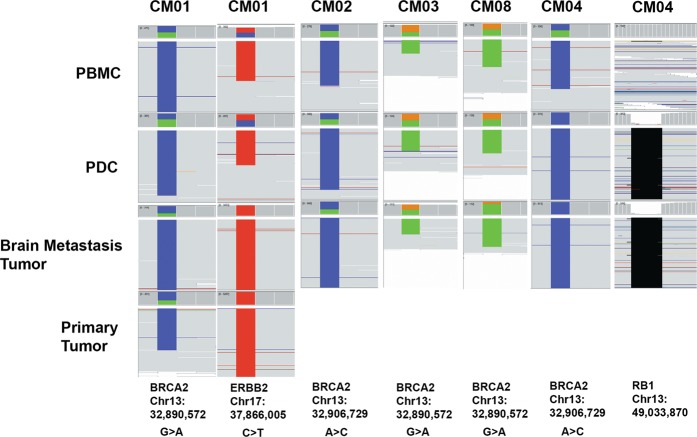
Germline analysis of PDCs visualized in Integrated Genomics Viewer. Exome-seq data for CM01, 02, 03, and 08-PDCs revealed retention of germline mutations at the same allelic ratios as seen in patient-matched PBMCs indicative of a normal cell of origin. The tumorigenic CM04-PDC demonstrated clear evidence of somatic mutations including LOH for *BRCA2* and a small deletion in *RB1* present in the patient CM and not in the corresponding PBMCs

Further analysis of exome-seq data revealed the retention of germline mutations in CM01, CM02, CM03, and CM08-PDCs with the same allelic ratios as seen in patient-matched peripheral blood mononuclear cells (PBMCs) (Fig. 2). Specifically, heterozygous germline mutations in *BRCA2* and/or ERBB2 were observed. In contrast, CM04-PDC demonstrated clear evidence of somatic mutations including loss of heterozygosity (LOH) for *BRCA2* and a small deletion in *RB1* present in the patient CM and not in the corresponding PBMCs. LOH towards the mutant allele occurred in *ERBB2* and *BRCA2* only in the patient metastases of CM01 (including the primary breast cancer), CM02, CM03, and CM08 but not in their corresponding PDCs (Fig. 2). The normal-like genome in CM01, CM02, CM03, CM08-PDCs is consistent with their observed senescence in late teen passage numbers. CM04-PDC, however, did not display any evidence of senescence indicating that these are immortalized cells. Sequencing data from CM01-PDC-X (exome and RNA) revealed that >98% of sequencing reads aligned to the mouse genome, suggesting that this cell line is likely derived from mouse stroma. However, for CM01-PDC-X, only reads that aligned uniquely to the human genome were analyzed. The Xenome tool was used to filter the mouse reads.

**Fig. 3:**
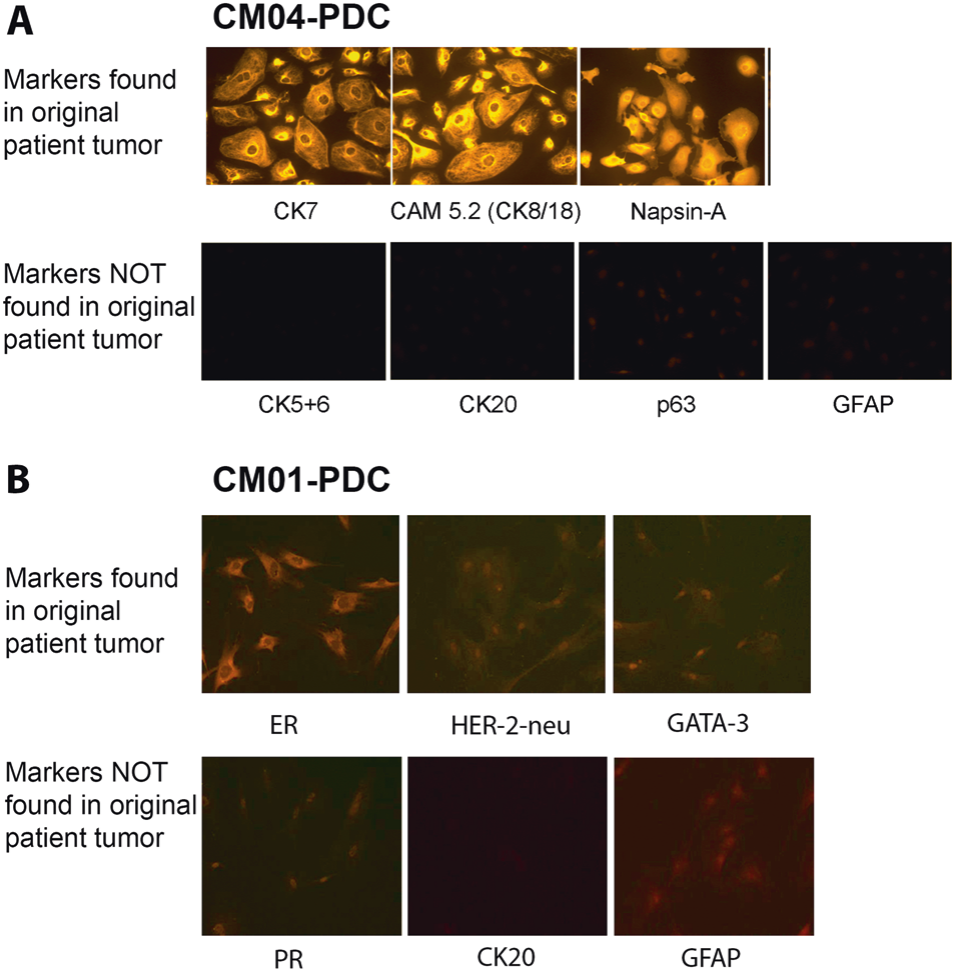
Immunofluorescence staining of PDCs for clinical markers found in the patient’s original CM tumor, and astrocyte marker GFAP. **a** Based on the patient pathology report, the tumorigenic CM04-PDC had positive staining of diagnostic markers consistent with a SCLC diagnosis. **b** Non-tumorigenic CM01-PDC, which was derived from a breast cancer CM, was negative for all of the patient’s clinical markers. Pathological scoring of these markers for all PDCs can be found in Supplementary Table 2

Next, we performed immunofluorescence to stain for diagnostic markers found in the original patient CM tumor as indicated in each patient’s pathology report **(**Suppl Table 2). Only CM04-PDC exhibited staining patterns consistent with its patient tumor, and demonstrated SCLC characteristics through positive staining of CK7, CAM 5.2, and Napsin-A, and negative staining for CK5/6, CK20, and p63 (Fig. 3a). In addition, both PDCs were also negative for GFAP, ruling out reactive astrocytes as the source of the cells. Conversely, the other PDCs were negative for all clinical markers found in the patient, regardless of whether they were positive or negative (Fig. 3b, Suppl Fig. 2). Taken together, these data demonstrate that there was a predilection for isolating stromal cells over tumor cells from surgical specimens, whereby CM01, CM02, CM03, and CM08-PDCs are deemed non-neoplastic cells derived from the CM tumor microenvironment and will be referred to as CNS Metastasis-associated stromal cells (cMASCs); CM01-PDC-X is an immortalized mouse stromal cell line, and CM04-PDC is an immortalized and transformed tumor cell line derived from a SCLC CM.

### cMASCs resemble cancer-associated fibroblasts derived from a common cell in the CM tumor microenvironment

**Fig. 4:**
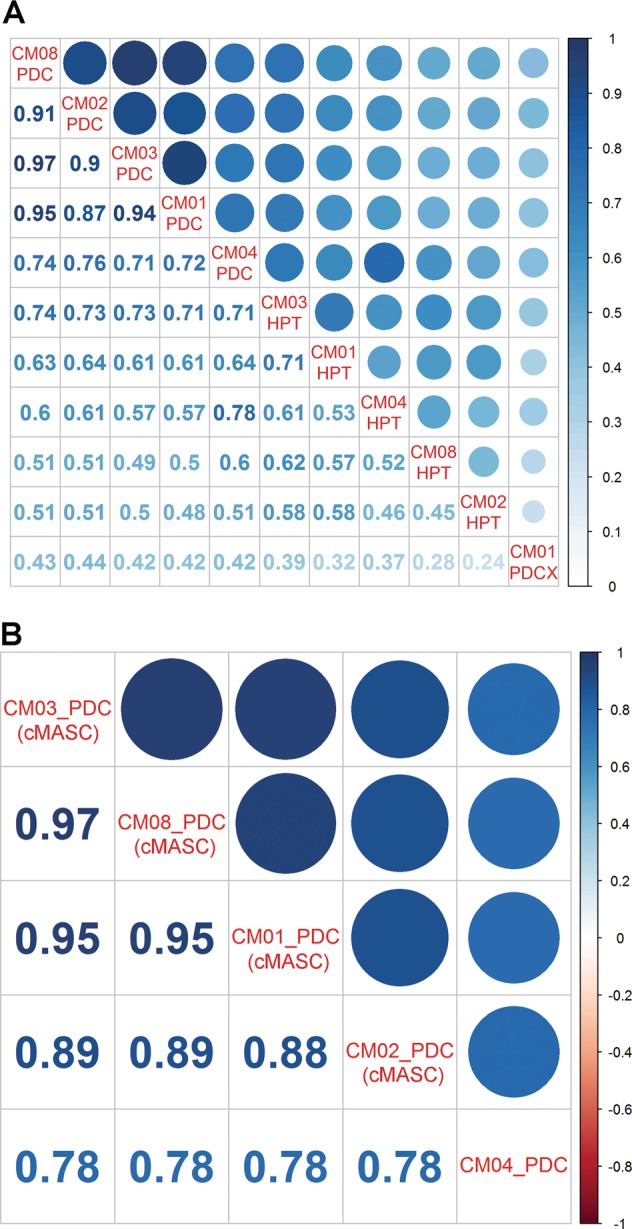
PCC matrix for comparison of PDCs/PDC-X and their matching human patient CM tumor (HPT). **a** PCC matrix of normalized FPKM values from RNA-seq data. Non-tumorigenic CM01, 02, 03, and 08-PDCs (cMASCs) show high degree of similarity with each other (0.87–0.97), but were dissimilar to the tumorigenic CM04-PDC (0.71–0.76) and their HPT (0.51 – 0.73). CM01-PDC-X displayed the least amount of similarity to any of the other PDCs owing to its murine host and origin. **b** PCC matrix utilizing normalized methylation β-values from 450K Illumina Methylation BeadArrays. CM04-PDC is the least similar to other PDCs with coefficients equal to 0.78. Methylation data for CM01-PDC-X is not available

To further investigate the molecular and cellular characteristics of PDCs and PDC-X, and to examine the potential cellular origin of cMASCs, we performed RNA-seq, DNA methylation profiling, and immunocytochemical analysis. We compared the global gene expression and DNA methylation profiles of each cell line along with their original human patient tumors (HPT). For this, we plotted normalized FPKM values from RNA-seq data or normalized β-values from 450K Illumina Methylation BeadArrays in a Pearson Correlation Coefficient (PCC) Matrix. Strikingly, the 4 normal human PDCs/cMASCs showed a high degree of correlation ranging from PCC 0.87–0.97 for RNA-seq (Fig. 4a) and 0.88–0.97 for DNA methylation (Fig. 4b) and the correlation to their matching HPT was much lower (PCC 0.51–0.73). In contrast, CM04-PDC correlated to a much lesser degree with cMASCs (CM01, CM02, CM03, and CM08), ranging from PCCs 0.71–0.76 for RNA-seq and 0.78 for DNA methylation **(**Fig. 4a, b). CM04-PDC had the highest correlation with its HPT at 0.78. This is remarkable considering that cells were isolated from four different CM patient tumors (CM01, CM02, CM03, and CM08) with widely different tumor pathologies and tumor characteristics (Suppl Table 1). These data are strong support for the notion that cMASCs are derived from a common cell of non-tumorigenic origin. CM01-PDC-X gene expression profile differed the most from the other cell lines (PCCs ranging from 0.44–0.47) including CM01-cMASC, and also its matching HPT (Fig. 4a). This finding emphasizes the impact of the host tumor microenvironment on observed expression profiles. We therefore postulate that cMASCs resemble cancer associated fibroblasts (CAFs), which likely did not arrive embedded in a metastatic tumor embolus to the brain, but rather were isolated from a common cell of origin within the tumor microenvironment of CM or drawn from circulating mesenchymal precursor cells.

**Fig. 5:**
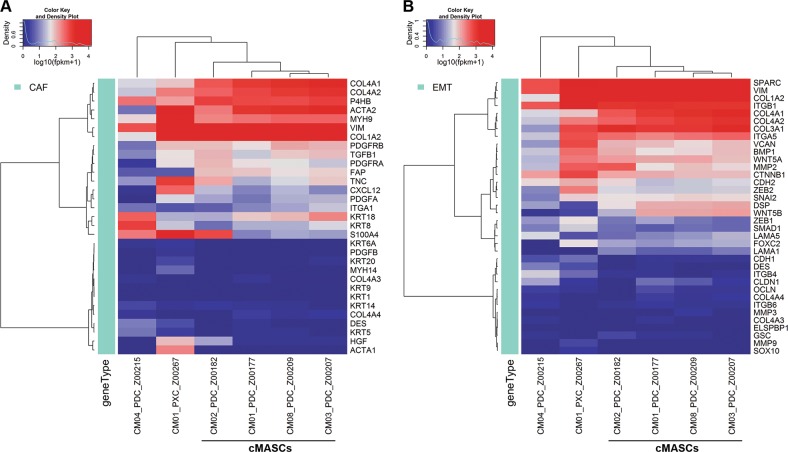
Hierarchical clustering analysis of PDCs for expression of CAF and EMT associated-genes. **a** A list of 31 CAF associated-genes was used for clustering analysis of PDCs. The dendrogram showed the similarity of the non-tumorigenic PDCs and confirmed their CAF-like nature with upregulation of *ACTA2*, *MYH9*, and *COL4A1*. CM04-PDC does not show this CAF expression signature. **b** A dendrogram demonstrated an EMT signature for CM01, 02, 03, and 08-PDCs evidenced by upregulation of *COL3A1*, *COL1A2*, *COL4A1*, *ITGB1*, *SPARC*, *ITGA5*, *VIM*, and low expression of *CDH1* and *OCLN*. CM04-PDC does not display this EMT signature

**Fig. 6:**
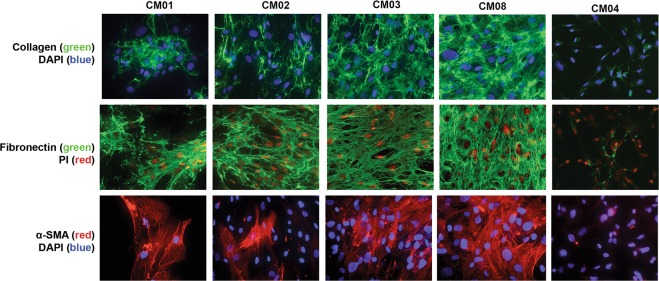
Immunocytochemical staining of cMASCs for ECM proteins collagen and fibronectin, and CAF marker α-SMA. DAPI or propidium iodide (PI) were used to visualize the nucleus. CM01, 02, 03, and 08-PDCs (cMASCs) showed abundant and intricate lattices of collagen and fibronectin and α-SMA staining was evident in a subpopulation of these cMASCs. By contrast, CM04-PDC had virtually no evidence of α-SMA, collagen or fibronectin expression

To more closely examine the notion that cMASCs resemble CAFs, we interrogated a set of 31 gene expression markers known to be closely associated with CAFs. Hierarchical clustering analysis of these genes in the 6 cell lines demonstrated strong expression of CAF genes (Fig. 5a), including alpha smooth muscle actin (*ACTA2*), myosin 9 (*MYH9*), and collagen type IV alpha 1 (*COL4A1*) in CM01 (cMASC and PDC-X), CM02, CM03 and CM08-cMASCs but not CM04-PDC. Interestingly, immunocytochemical staining revealed that only a subpopulation of cMASCs were positive for α-smooth muscle actin (SMA) (Fig. 6). These cells also expressed intermediate levels of additional CAF markers including platelet-derived growth factor receptor beta (*PDGFRβ*), transforming growth factor beta 1 (*TGFβ1*), and fibroblast activation protein (*FAP*) (Fig. 5a). Furthermore, it is demonstrated that CM01, CM02, CM03, CM08-PDCs and CM01-PDC-X cells expressed markers of epithelial to mesenchymal transition (EMT, a common feature of CAFs) as evidenced by upregulation of *COL3A1*, *COL1A2*, *COL4A1*, *ITGB1*, *SPARC*, *ITGA5*, *VIM*, and low expression of *CDH1* and *OCLN* (Fig. 5b). As well, abundant and intricate matrices of collagen and fibronectin was evident uniquely in PDCs (CM01, CM02, CM03, CM08) (Fig. 6).

**Fig. 7:**
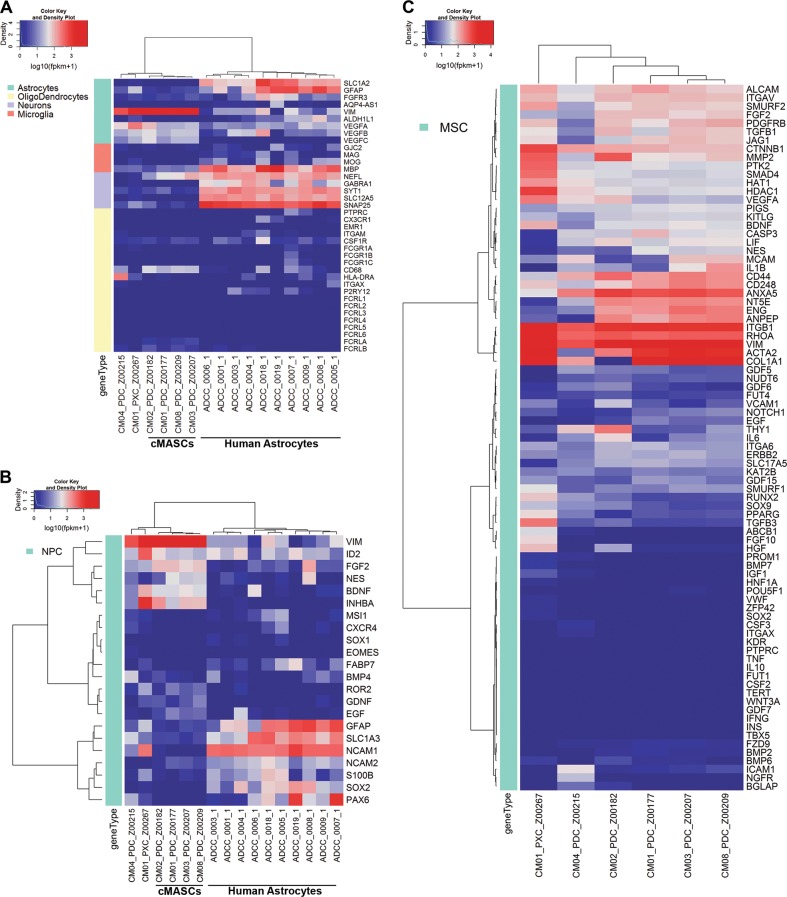
Hierarchical clustering analysis of RNA-seq expression data for neural and stem cell genes. **a** Clustering analysis using a set of glial lineage markers showed that PDCs did not match the gene expression profiles of fully differentiated glial cells or neurons as they were clearly separated from the human astrocytes (designated by ADCC [17]) included in the analysis. **b** PDCs clustered separately from human astrocytes when analyzed with a set neural progenitor cell markers. **c** Non-tumoral PDCs showed concerted expression of mesenchymal stem cell markers *ENG*, *NT5E*, *ITGB1*, *ACTA2*, *COL1A1*, *CD44*, and *ANXA5*, but did not express markers of MSC differentiation such as *SOX9* or *BMP7*. Additionally, expression of the pericyte marker *CD248* was observed

In contrast, CM04, the tumorigenic PDC, expressed much higher levels of epithelial markers (*KRT8*, and *KRT18*) (Fig. 5a), showed much lower levels of expression for CAF-specific genes, did not display strong evidence of EMT (Fig. 5b), and clustered separately from the other lines in hierarchical clustering analysis of various gene lists (Figs. 5a, b, 7a–c). Notably, CM01-PDC-X and CM02-PDC expressed the lowest levels of *KRT18*. In addition, CM04-PDC had virtually no evidence of α-SMA, collagen or fibronectin expression (Fig. 6). CM01-PDC-X also differed from the other PDCs, however, it still displayed hallmarks of EMT and CAF association **(**Fig. 5a, b**)**. As CM01-PDC-X was quite distinguishable from other PDCs and derived from a PDX, it was not given the cMASC designation.

### cMASCs cell of origin

There is varying evidence supporting the genesis of CAFs from resident fibroblasts, bone marrow-derived mesenchymal stem cells (MSCs), or cancer cells that undergo epithelial or endothelial-mesenchymal transition [10]. In order to identify the cell type of origin for CM01, CM02, CM03, CM08-cMASCs and CM01-PDC-X, RNA expression for each of these lines was normalized to the RNA expression values of CM04-PDC (tumor cell line) and the NextBio Body Atlas database [16] was queried for enriched cell type similarities. Among the top 10 highest correlated cell types were mesenchymal stem cells and other cells of mesodermal origin (Suppl Fig. 3). Most interestingly, CM01-PDC-X had a strong association with breast cancer stromal cells. Astrocytes, which arise from multipotent neural stem cells through the generation of lineage-committed glial restricted progenitor cells, were also identified as a potential cell type of origin (Suppl Fig. 3).

Therefore, we also examined gene expression of glial lineage markers (astrocytes, oligodendrocytes, microglia), neuronal lineage markers (neurons and neural progenitor cells), and mesenchymal stem cell markers. We incorporated known data from human astrocytes isolated from cadaver brain previously published in ref. [17] as a comparison. Hierarchical clustering results are represented as heatmaps of the normalized FPKM expression values **(**Fig. 7a–c**)**. Meaningfully, the data demonstrated that cMASCs did not match the gene expression profiles of fully differentiated glial cells or neurons as they were separated quite significantly from the human astrocytes included in the analysis (Fig. 7a). Furthermore, cMASCs did not express GFAP by immunocytochemistry (Fig. 2). In addition, cMASCs did not ubiquitously express important markers associated with neural progenitor cells **(**Fig. 7b). However, they expressed many MSC specific markers including *ENG*, *NT5E*, *ITGB1*, *ACTA2*, *COL1A1*, *CD44*, and *ANXA5*, but did not express markers of MSC differentiation such as *SOX9* or *BMP7* [18] (Fig. 7c). In addition, cMASCs expressed the pericyte marker *CD248* (Fig. 7c). In the case of each of these gene expression lists, CM04-PDC and CM01-PDC-X were distinguishable from cMASCs and did not appear to have MSC lineage markers. Collectively, the presented data indicate that cMASCs resemble CAFs that may have been derived from a population of brain MSCs exhibiting hallmarks of EMT. Moreover, the human sequence analyzed from CM01-PDC-X suggested that there may have been a very small subpopulation of human cells amongst the largely murine background of this cell line. This could also be an indication of the mouse stroma replacing and modifying the human stroma originally implanted in mice.

### Human cMASCs inhibit tumor growth in vivo

**Fig. 8:**
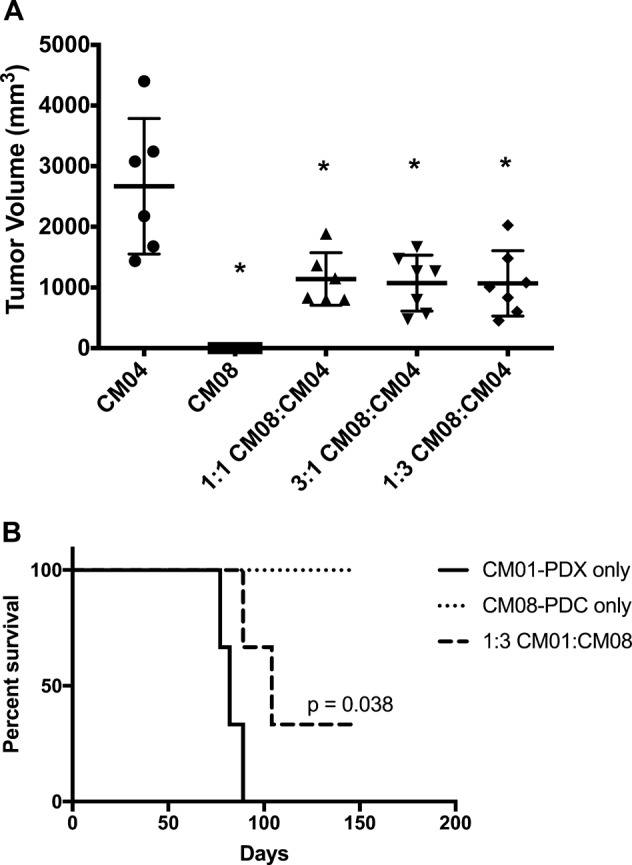
cMASCs limit tumor growth in vivo. **a** CM04-PDC and CM08-PDC were xenografted into the right flank of mice either alone, or combined at ratios of 1:1, 3:1 or 1:3. In vivo tumor volume measurements after 40 days are graphed as a box and whisker plot. Combination of CM04-PDC and CM08-PDC in different ratios led to smaller tumors in mice (*p* value < 0.05). **b** Kaplan–Meier survival curve of mice implanted intracranially with CM01-PDX alone or combined with CM08-PDC in 3:1 ratio. Combination of CM08-PDC with CM01-PDX increased mean survival by 13.8 days (*p* = 0.038)

To determine the possible contribution of cMASCs to tumor cell growth and confirm the tumorigenicity of CM04-PDC, we performed a series of an in vivo experiments to compare the growth of tumor cells alone and after combining with cMASCs. In the first of these experiments we combined CM08 cMASC with CM04-PDC. Cells were mixed in 1:1, 1:3, and 3:1 ratios of CM08:CM04 and injected into the flanks of 6–7 mice per group. The data confirmed that CM04-PDC is tumorigenic in mice and CM08-PDC was non-tumorigenic (Fig. 8). At 40 days post injection, the mean size of CM04 tumors was 2668 mm^3^. However, the combination of CM08-PDC with CM04-PDC led to statistically significant inhibition of tumor growth by about two and half-fold. The following was the mean tumor size 40 days post injection in the mixed groups: 1140 mm^3^ (1:1, *p*-value = 0.0108), 1072 mm^3^ (1:3, *p*-value = 0.0062) and 1069 mm^3^ (3:1, *p*-value = 0.0052) (Fig. 8a). Next, we combined CM08-PDC with CM01-PDX in a 3:1 ratio and implanted into the brains of 3 mice per group (Fig. 8b). Average survival of the mixed group was significantly higher than the tumor only group, 96.5 days vs. 82.7 days, respectively (*p* value = 0.038). Lastly, we combined CM08-PDC with the MDA-MB-231BR (231-BR, brain seeker clone of parental MDA-MB-231 cells [19]) cell line in a 3:1 ratio. The combination of CM08-PDC and 231-BR also reduced the mean tumor size from 1937 mm^3^ in the 231-BR only tumors to 1552 mm^3^ (*p*-value = 0.17) in the mixed tumors (Suppl Fig. 5A). While the results of 231-BR did not achieve statistically significance, 3 admixed tumors had a mean tumor size (1178 mm^3^) significantly smaller than control animals, indiciating that these results are consistent with the overall findings. Collectively, these data demonstrate that cMASCs can inhibit tumor growth.

**Fig. 9:**
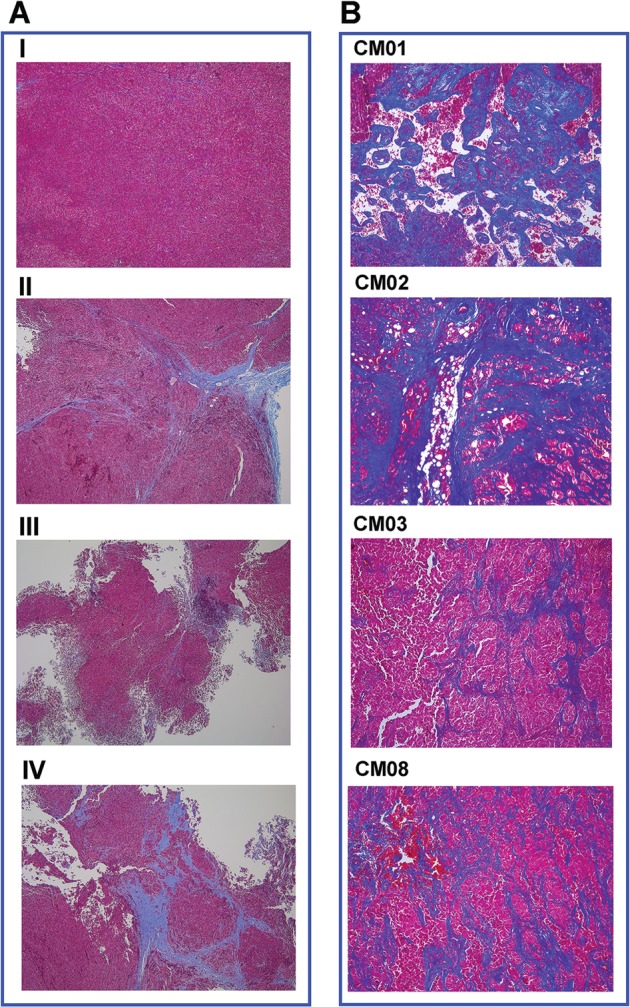
Trichrome staining reveals desmoplasia in cMASC admixed tumors in vivo and in human samples. **a** Formalin fixed paraffin embedded whole sections of mouse tumors from control CM04-PDC only tumors and mixed tumors of CM04:CM08 co-implantation. Panel I: CM04-PDC only; Panel II: 1:1 CM08-cMASC:CM04-PDC; Panel III: 3:1 CM08-cMASC:CM04-PDC; Panel IV: 1:3 CM08-cMASC:CM04-PDC. Staining revealed a fibrotic response (desmoplasia), indicated by deposition of abundant collagen matrices (blue staining) only in mixed tumors. **b** Trichrome staining of patient tumors for CM01, 02, 03, and 08 also showed an extensive desmoplastic response (blue staining)

Since cMASCs displayed abundant collagen deposition in vitro (Fig. 6), whilst CM04-PDC did not, we performed trichrome staining on formalin fixed paraffin embedded whole sections of mouse tumors taken from the three experiments combining tumor cells with cMASCs to investigate whether collagen could also distinguish cMASCs in vivo. Analysis of the trichrome staining revealed a stronger fibrotic (desmoplastic) response preferentially in the tumor/cMASC combined tumors (Fig. 9a, Suppl Fig. 5B, C). Moreover, the trichrome staining of the patient tumors of CM01, CM02, CM03, and CM08 (CM04 was not available) revealed extensive desmoplasia (Fig. 9b). Although we were not able to obtain the sections from the patient tumor of CM04, the lack of collagen fibers in both the cell culture and tumors in mice strongly supports the notion that cMASCs are contributing to desmoplasia in CM. These data also suggest that such an intense production of a collagen-rich extracellular matrix by mesodermal-derived non-tumorigenic cells within the tumor microenvironment may provide a physical barrier/plug preventing the further growth and spreading of tumors cells. Clinically, the slowest growing metastases tend to be squamous cell carcinoma CMs, which are the most fibrotic (desmoplastic) of the brain tumors.

## Discussion

CAFs are the most prominent cell type within the tumor microenvironment of many cancers, most notably breast, prostate, and pancreatic carcinoma [10]. Despite burgeoning literature on the complexities of tumor-stromal interactions, the role of CAFs in tumor biology, and novel therapeutics, there remains a dearth of data on the ultimate contribution of CAFs to the tumor microenvironment of CM. Although CAFs have been previously identified in human and mouse CM, their functional analyses have not gone beyond immunohistochemistry [20]. Moreover, it is becoming clear that the taxonomy of CAFs varies both inter and intra-tumorally [10].

Still, several studies have shown that CAFs can arise from a myriad of cell types including by trans-differentiation of resting resident fibroblasts or pericytes within the tumor microenvironment [10]. CAFs could also arise from bone marrow-derived MSCs, or from normal epithelial or transformed cells via epithelial or endothelial to mesenchymal transition, among other possibilities. While in systemic metastasis resident CAFs originate primarily by activation of local fibroblasts by cancer-derived growth factors, fibroblasts are not likely the origin of cMASCs since they are not resident cells of the brain. We postulate that most likely cMASCs represent a unique population of mesodermal CNS-residing cells that have been trans-differentiated under the influence of the microenvironment of the infiltrating metastatic tumor cells. Importantly, our gene expression analysis has effectively ruled out cMASCs as being derived from a glial or neuronal cell lineage.

MSCs are multipotent stromal cells that can differentiate into a variety of cell types, including: osteoblasts (bone cells), chondrocytes (cartilage cells), myocytes (muscle cells) and adipocytes (fat cells). The mesodermal characteristics of cMASCs demonstrated by NextBio Body Atlas database analysis also strongly supports a MSC heritage. Recently, a better understanding of the native location of MSCs has been sought. Many studies have designated microvascular pericytes as at least one class of tissue resident MSCs. cMASCs expressed the MSC markers *NT5E*, *ALCAM*, and *ENG* and also expressed *CD248*, a known pericyte marker (Fig. 7c).

Lojewski *et al*. found marked similarities between human brain-derived pericytes and bone marrow-derived mesodermal stem cells. Pericytes, which can be isolated from the hippocampus and subcortical white matter were easily expandable in culture and expressed many surface markers in common with MSCs. They also showed a preferred propensity for mesodermal differentiation characteristics and eschewed those of the neuronal ectoderm [21]. This study supports our findings to the extent that it points to the possibility that cMASCs are an intrinsic population of bone-marrow derived MSCs that manifest as pericytes in the brain tumor microenvironment. Still, further studies are ongoing in our laboratory to functionally corroborate the cellular origin of cMASCs. An important clinical implication to these findings lies in the fact that bone marrow-derived MSCs are of therapeutic interest in a variety of neurological diseases. MSCs and their secretome are being proposed as strong candidates mediating repair from CNS injury [22] and our data suggest that cMASCs may be of therapeutic importance in CM.

Indeed, many cancers are associated with desmoplasia, a common fibrotic state, characterized by an accumulation of type I and III collagens, and accompanied by increased degradation of type IV collagen [23, 24]. Of note, tumor desmoplasia has been associated with poor prognosis of cancers [25], tumor progression and invasiveness, and increased chemotherapy resistance through decreasing drug uptake [26, 10]. This includes the promotion of EMT by collagens in the ECM secreted by CAFs [27], which could lead to increased proliferative potential of tumor cells [28]. However, the desmoplastic reaction can be context dependent and also thought to represent a host defense mechanism, similar to wound healing and tissue regeneration, to repair or hopefully impede the conversion of a neoplastic lesion into invasive carcinoma [12, 13, 29]. The cellular component of the desmoplastic stroma of cancers is composed primarily of CAFs. The notion that desmoplasia is a host protective mechanism is more consistent with our findings and with recent findings in pancreatic cancer, where it was demonstrated that depletion of myofibroblasts (activated fibroblasts) using compound genetic mouse models of pancreatic ductal adenocarcinoma (PDAC) led to aggressive tumors with diminished animal survival [30]. In concert with this finding, reduction of fibrosis did not increase the efficacy of gemcitabine in PDAC. Fewer myofibroblasts in human PDAC also correlated with reduced patient survival and clinical trials targeting stromal myofibroblasts in human PDAC resulted in an apparent paradoxical accelerated disease progression, halting the clinical trials [31].

Our study is of significance because, for the first time, a cMASC with CAF features derived from the tumor milieu of CM specimens has been isolated and characterized. We demonstrated by gene expression profiling that cMASCs likely originate from mesodermal cells such as MSCs, but most importantly they function to inhibit/restrict tumor growth by possibly mounting a desmoplastic reaction. In addition to the establishment of cMASCs, the establishment of CM04-PDC (a tumorigenic cell line from a patient with SCLC to brain) represents an excellent new model system for future studies in SCLC CM. A remarkable finding in our study was related to how similar the 4 cMASC lines were to each other, in spite of the fact they came from different individuals with very unique tumor profiles representing both lung and breast CM. This therefore suggests that cMASCs represent a common feature of the CM tumor cells to go along with cMASCs, however, the predilection for the isolation of cMASCs made this difficult to achieve. Perhaps, modifying tissue culture media or cell sorting can circumvent this in the future.

In summary, we demonstrated the isolation, molecular characterization and functional role of human derived cMASCs, a subpopulation of cells in the microenvironment of CM that have tumor inhibitory functions. The data offer evidence that the matrix produced by human derived cMASCs may constitute a protective barrier, in the host’s attempt to limit further growth of the tumor. Unfortunately, the dismal prognosis of CM suggests that this desmoplastic reaction is not sufficient to constrain the growth of the CM. Moreover, extensive desmoplasia seen in patient tumors may also lead to other sequelae such as inflammation, and adversely trigger tissue remodeling. In addition, desmoplasia may lead to increased tumor volume, which would consequently increase compression of the brain, potentially leading to increased intracranial pressure and earlier death than a tumor without desmoplasia. Therefore, further studies should also be focused on understanding the molecular players of cMASC-mediated desmoplasia within the tumor microenvironment of CM in order to develop methods of pharmacological control that preferentially target its potentially negative sequelae while preserving its anti-tumor growth role.

## Materials and methods

### Tissue acquisition

Patients with brain metastases secondary to breast or lung cancer were screened by the surgeon (SAT, MB) and consented for tissue collection at Geisinger Health System (Danville, PA) or University Health Network (Toronto, Canada) under an IRB approved protocol. During craniotomy, tumors were excised and excess viable tumor specimen was collected, minced into 2–3 mm^3^ pieces, and placed into 50 ml sterile Falcon tubes (Corning, MA) containing DMEM/F12 medium (Gibco, MA) and shipped for next day delivery. Our laboratory (BS) received fresh tissue for generation of patient-derived cell lines (PDCs). An additional 20–30 mg piece of tumor tissue was flash frozen for later use in genomic profiling. A separate piece of fresh tissue was used for implantation into animals for development of PDXs. Annotated, de-identified clinical data was made available for each patient. Formalin fixed, paraffin embedded (FFPE) tissue samples from the metastatic tumors were collected, when available.

### Establishment of primary cell cultures

Fresh tumor tissue was aseptically minced into fine pieces (1–5 mm^3^) and placed into 25 cm^2^ culture flasks (Corning, MA) for establishment of PDCs. Cells were cultured in Advanced DMEM/F12 medium (Gibco, MA), supplemented with 10% FBS, 2 mM L-glutamine, 10 ng/mL epidermal growth factor, 5 U/mL penicillin, 5 μg/mL streptomycin (all obtained from Gibco, MA), 2.5% BD NuSerum IV (Corning, MA), and 10 ng/mL Cholera toxin (Sigma-Aldrich, MO). Primary cultures were initially dissociated with either StemPro Accutase, or a 0.05% Trypsin-EDTA solution (Gibco, MA), cultured at 37 °C, and passaged once cells reached approximately 70% confluency. In one instance (CM01), a cell line from a PDX was established as described above, but rather referred to as a patient-derived xenograft cell line (PDC-X). PDC/PDC-Xs ranging from passages 8–12 were used for all subsequent experiments.

### Immunofluorescence

#### Staining for confirmation of clinical histological characteristics

Cells were seeded on poly-D-lysine-coated coverslips overnight. Cells were fixed in 4% paraformaldehyde (Sigma-Aldrich, MO) and permeabilized with 0.1% Triton-X 100 (Sigma-Aldrich, MO). Nonspecific binding was blocked by incubating cells in 5% w/v BSA in PBS-T (Sigma-Aldrich, MO) for 30 min at room temperature. Cells were incubated with primary antibodies diluted in blocking buffer overnight at 4 °C (Suppl Table 3). Post incubation of primary antibodies, cells were washed and incubated with Alexa-Fluor 568-conjugated secondary antibodies (Life Technologies, CA) diluted 1:700 in blocking buffer for one hour at room temperature [32]

#### Collagen 1, fibronectin, and α-smooth muscle actin (SMA) staining

Cells were either fixed for 30 min in cold 100% methanol at −20 °C (for detection of fibronectin and α-SMA) or in 4% paraformaldehyde at room temperature (for detection of collagen type I). After blocking for one hour with 1% normal goat serum (Gibco, MA) in PBS, the parallel cultures were then incubated for 1 h (at room temperature) with either 1 µg/ml of rabbit polyclonal antibody to fibronectin (sc-9068 - Santa Cruz Biotechnology, CA), 5 µg/ml goat polyclonal antibody to collagen type I (AB758 - Millipore, MA), or with 5 µg/ml rabbit polyclonal antibody recognizing α-SMA (ab14106, Abcam, MA). Parallel cultures were then incubated with the appropriate fluorescein-conjugated goat anti-rabbit and rabbit anti-goat, or rhodamine-conjugated goat anti-rabbit secondary antibodies.

Cell nuclei were counter-stained with either propidium iodide (Santa Cruz Biotechnology, CA) or 4,6-diamidino-2-phenylindole (DAPI, Life Technologies, CA). All cultures were then rinsed in PBS, mounted with ProLong Gold Antifade, and analyzed under a fluorescence microscope.

#### Trichrome staining

The trichrome stain was used for histological visualization of collagenous connective tissue fibers [33, 34].

### Nucleic acid extraction

See Supplementary Methods

### Exome sequencing

See Supplementary Methods

### RNA sequencing

See Supplementary Methods

### Analysis

Sequence data was analyzed using TGen’s pipeline which follows best practices for genomics data analyses [35, 36]. HiSeq 2500 generated BCL files were converted to FastQ files (raw sequence) using the bcl2fastq 1.8.4 tool [http://support.illumina.com/sequencing/sequencing_software/bcl2fastq-conversion-software/downloads.html]. Exome reads were aligned against Human GRCh37.74 reference genome using BWA-MEM [37], while RNA-Seq reads were aligned to the aforementioned reference genome using STAR [38].

Aligned exome reads were used to identify copy number aberrations (CNAs), germline, and somatic variants. Copy number analysis was performed with TGen’s CNA pipeline using a custom algorithm to capture copy number losses and gains along with neutral copies (https://github.com/tgen/tCoNuT). Log2 ratio of tumor or cell line DNA relative to each patient’s germline control was outputted, and log2 ratios between −1 and+1 were filtered out of further analyses.

Germline variants were visualized in Integrated Genomics Viewer. Somatic variant calling was performed using, Seurat [39], Strelka [40], and MuTect [41]. Somatic calls from the three variant caller tools were merged using a custom TGen script, and the VCF output file annotated with SnpEff tool.

### NextBio body atlas analysis

See Supplementary Methods

### DNA methylation analysis with 450 K beadarray [42]

See Supplementary Methods

### In vivo Studies

A total cell suspension of 5 × 10^6^ cells was mixed with a 1:1 v/v of growth factor reduced Matrigel (Corning, MA) and injected subcutaneously into the right flank of female, 6–8 week old CIEA NOG mice (Taconic, NY) with a 1 cc tuberculin syringe and 25 G needle. 6 animals per group was estimated to give >90% power to detect a 25% difference. 6 (Group I, III) or 7 (Group II, IV, V) mice per group were injected. Tumor volume and animal weight were measured every other day with calipers and tumor volume was calculated using the following formula: (LxWxWx0.5), where the smaller of the two dimensions is used twice. When tumor volume reached a maximum of 2000 mm^3^, the mouse was euthanized.

For the intracranial injection, mice (*n* = 3 per group) were anesthesia with isoflurane and placed into stereotaxic frame (Stoelting). A 5–7 mm skin incision was made and a burr hole drilled 1–2 mm lateral to the midline and 2–3 mm vertical from the bregma suture. An automated injection system was used to slowly inject 2–3 µl of 10^4^ cells into the brain parenchyma. No randomization or blinding was performed. The animal studies were conducted under IACUC approval at the University of Arizona and University of Southern California Vivaria.

## Supplementary information

Supplementary Methods

Supplementary Figure Legends

Supplementary Figure 1

Supplementary Figure 2

Supplementary Figure 3

Supplementary Figure 4

Supplementary Figure 5

Supplementary Table 1

Supplementary Table 2

Supplementary Table 3
