# Supplementary material for: Isolation and characterization of patient-derived CNS metastasis-associated stromal cell lines
Source: Oncogene. 2019 Jan 30;38(21):4002–14. doi: 10.1038/s41388-019-0680-2 (PMC6756000; doi:10.1038/s41388-019-0680-2)
Supplement: Supplementary file 1 — Supplementary Methods [file 41388_2019_680_MOESM1_ESM.docx]

**Supplementary Methods:**

**Nucleic Acid Extraction**

Tissue: 10-25mg pieces of flash-frozen tumor tissue were homogenized in the Bullet Blender Homogenizer (Next Advance, NY) for 5 minutes, at full speed, with a mixture of 0.9-2.0mm RNase-free stainless steel beads. Homogenates were then passed over a QIAshredder homogenizing column (Qiagen, CA) to remove any remaining particulate matter. Genomic DNA and total RNA were extracted simultaneously from each lysate using the AllPrep DNA/RNA Mini kit (Qiagen, CA) according to the manufacturer’s recommendations. RNA was eluted in 50μl ultra-pure H_2_O and stored at -80ºC. DNA was eluted in 100μl EB buffer and stored at -20ºC.

Whole Blood: 5-10ml samples of frozen whole blood were used to extract genomic DNA from peripheral blood mononuclear cells (PBMCs) with the QIAamp DNA Blood Maxi kit (Qiagen, CA) according to the manufacturer’s recommendations. DNA was eluted in 600μl AE buffer and stored in small aliquots at -20ºC.

Cultured Cells: Cells were plated at a density of approximately 70% confluency and allowed to grow overnight. Genomic DNA was extracted with the Gentra Puregene Cell kit (Qiagen, CA) and total RNA was extracted with the RNeasy Mini kit (Qiagen, CA) according to manufacturer’s recommendations. DNA and RNA were eluted in T low E buffer or ultra-pure H_2_O respectively, and stored as described above.

Quantitation: Nucleic acids were quantified using a NanoDrop 1000 Spectrophotometer (ThermoFisher, MA).

**Exome Sequencing**

Libraries for sequencing were prepared using the SureSelectXT Target Enrichment System (Agilent, CA). The input amount of DNA was 1.0 μg, except for formalin-fixed paraffin embedded (FFPE) DNA, which used 3.0 μg. The samples were fragmented to an average size of 200 bp using the Covaris S2 system (Covaris, MA). The manufacturer’s recommended protocol for subsequent end repair, 3’ dA-tailing, and adaptor ligation was followed with the exception that the amount of adaptor oligo mix used was scaled down proportionally for DNA input amounts of 1.0 μg. Samples were then PCR amplified and cleaned up as per the protocol.

Hybridization/capture was accomplished with Human All Exon v5 (Agilent, CA) baits and 750 ng of prepped DNA. An 11 cycle indexing PCR and subsequent cleanup were performed following the manufacturer’s suggestions. Final library quality control was conducted using BioAnalyzer DNA 1000 chips (Agilent, CA) and the Qubit dsDNA High Sensitivity fluorometric assay (Invitrogen, CA). Equimolar pools of 16 libraries were created at a concentration of 5nM. Pools were subsequently diluted and clustered on the Illumina cBot using TruSeq Paired End Cluster Kit v.3 chemistry. Paired end sequencing for each pool was performed over 8 lanes on the Illumina HiSeq 2500 platform using TruSeq SBS v3 kits, for a total read length of 200bp. On average, 192 million read pairs were obtained per sample, resulting in 132X coverage.

**RNA Sequencing**

TruSeq RNA Sample Preparation v2 (Illumina, CA) kits were used for library preparation. One microgram total RNA input was used, and the manufacturer’s suggested protocol was followed. Subsequently, library preparation, QC was performed using BioAnalyzer DNA 1000 chips (Agilent, CA) and the Qubit dsDNA High Sensitivity fluorometric assay (Invitrogen, CA). Equimolar pools of 12 libraries were created at a concentration of 5nM. The pools were subsequently diluted and clustered on the Illumina cBot using TruSeq Paired End Cluster Kit v.3 chemistry. Paired end sequencing for each pool was performed over 4 lanes on the Illumina HiSeq 2500 platform using TruSeq SBS v3 kits, for a total read length of 166bp. On average, 53.8 million read pairs were obtained per samples, 95.73% of which were successfully mapped.

Gene expression estimates were calculated and normalized across samples using the modules of the latest version of the cufflinks suite tool. In brief, for each sample, STAR's alignment BAM file was used as input to the cufflinks module in order to estimates the genes and transcripts expressions as FPKMs values (Fragments Per Kilobase of exon per Million reads). Cufflinks' output file of FPKMs estimates got converted into a CXB binary file using the CuffQuant module to facilitate further data analysis and normalization of FPKMs in particular. All the CXB files, i.e. all FPKMs estimates from all the samples of interest, were then used to normalize the FPKM estimates across all the samples. This normalization was performed with the Cuffnorm module of the Cufflinks Suite tool. All the modules, CuffLinks, CuffQuant, Cuffnorm were used with default values and according to the author's recommendations.

**DNA Methylation Analysis with 450K BeadArray**

DNA Methylation analysis was performed using the Infinium HumanMethylation450 BeadChip Kit (Illumina, CA) as described in ^42^.

**NextBio Body Atlas Analysis**

RNA expression for CM01,2,3 and 8 PDCs was normalized to the RNA expression values of CM04-PDC. Normalized RNAseq data for CM01,2,3 and 8 PDCs was loaded into NextBio’s research platform (Illumina, CA) and the Body Atlas cell type library was queried for enriched cell type similarities ^16^.

**Data Deposition**

Sequencing data has been deposited in BioProject with accession number 510710.

**Statistical Testing**

Student’s t-test (two-sided and equal variance) or Wilcoxon signed-rank (two-sided) was performed and association was considered significant when P < 0.05 and indicated by an asterisk (*). Unless otherwise specified, error bars indicate the standard error of the main (s.e.m.). Power analysis was used to determine the sample size for *in vivo* studies.
