# Supplementary material for: Isolation and characterization of patient-derived CNS metastasis-associated stromal cell lines
Source: Oncogene. 2019 Jan 30;38(21):4002–14. doi: 10.1038/s41388-019-0680-2 (PMC6756000; doi:10.1038/s41388-019-0680-2)
Supplement: Supplementary file 2 — Supplementary Figure Legends [file 41388_2019_680_MOESM2_ESM.docx]

**Supplementary Figure Legends:**

**Suppl Fig 1.** CNA profile of patient-derived cell lines. CM01/02/03-PDCs displayed minimal copy number aberrations and do not resemble their patient tumor.

**Suppl Fig 2.** Immunofluorescence staining of PDCs for clinical markers found in each patient’s original CM tumor for CM02-PDC and CM03-PDC as reported in the patient’s pathology report. Pathological scoring of these markers for all PDCs can be found in **Supplementary Table 2**.

**Suppl Fig 3.** NextBio Body Atlas Analysis. RNA expression for CM01, 02, 03, 08-PDCs and CM01-PDC-X were normalized to the RNA expression values of CM04-PDC (tumor cell line) and the NextBio Body Atlas database was queried for enriched cell type similarities. Among the top 10 highest correlated cell types were mesenchymal stem cells and other cells of mesodermal origin.

**Suppl Fig 4.** Representative images from H&E staining of formalin fixed paraffin embedded whole sections of mouse tumors from control CM04-PDC only tumors and mixed tumors of CM04:CM08 co-implantation. A: CM04-PDC only; B: 1:1 CM08-PDC:CM04-PDC; C: 3:1 CM08-PDC:CM04-PDC; D: 1:3 CM08-PDC:CM04-PDC.

**Suppl Fig 5.** cMASCs limited tumor growth of 231-BR cells and increased desmoplasia. A) 231-BR alone (Group A), CM08-PDC alone (Group B) or 231-BR mixed with CM08-PDC (Group E) were implanted in the flank of mice. *In vivo* tumor volume measurements are graphed in a box and whisper plot. Combination of 231-BR and CM08-PDC led to smaller tumors. B) Trichrome staining of xenografted tumors. Panel I: 231-BR only; Panel II: 3:1 CM08-PDC:231-BR. C) Trichrome staining of intracranial implanted tumors. Panel I: CM01-PDX only; Panel II: 3:1 CM08-PDC:CM01-PDX.
