# Supplementary figures and images for: Isolation and characterization of patient-derived CNS metastasis-associated stromal cell lines
Source: Oncogene. 2019 Jan 30;38(21):4002–14. doi: 10.1038/s41388-019-0680-2 (PMC6756000; doi:10.1038/s41388-019-0680-2)

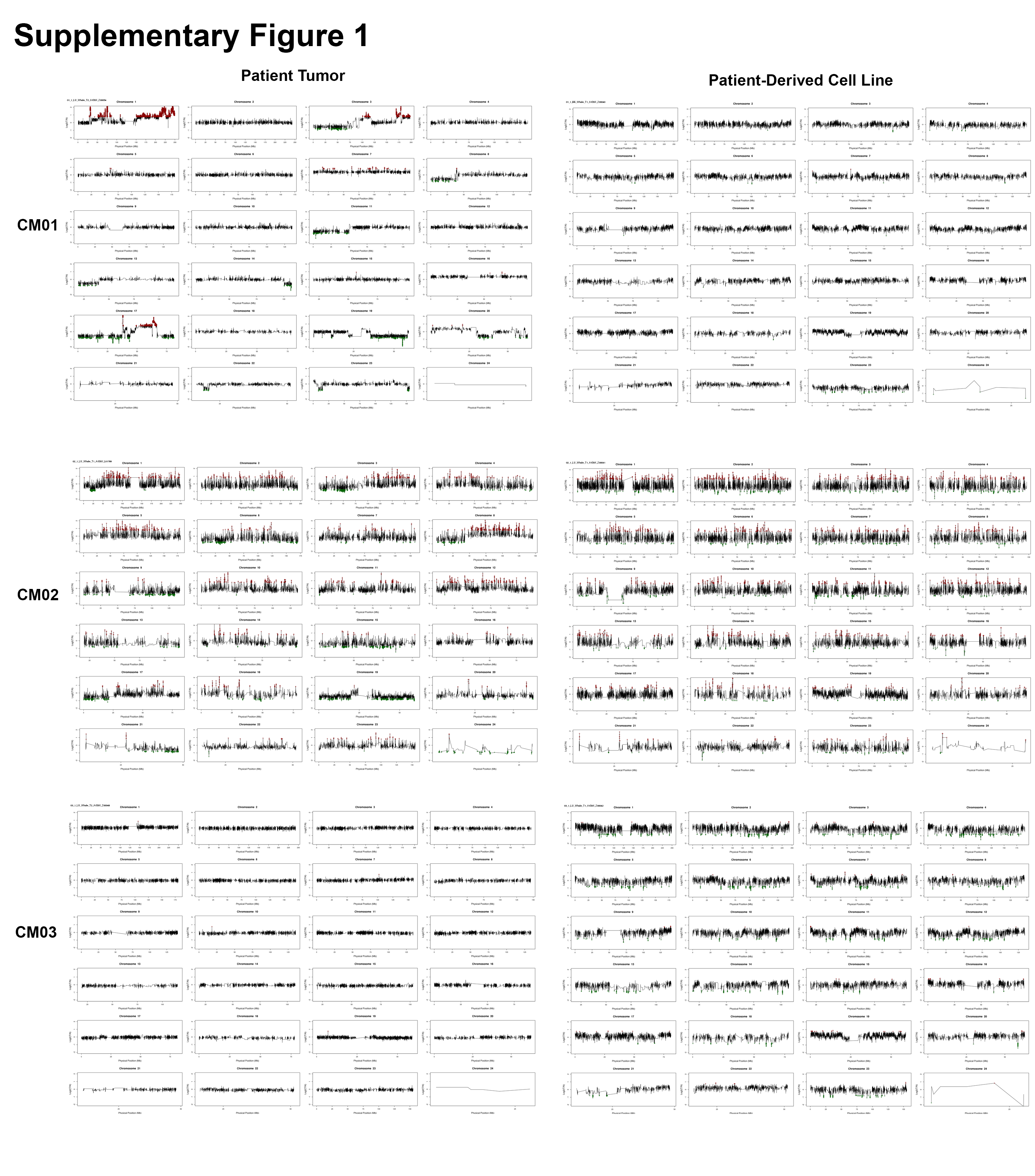

Supplement: Supplementary file 3 — Supplementary Figure 1 [file 41388_2019_680_MOESM3_ESM.tif]

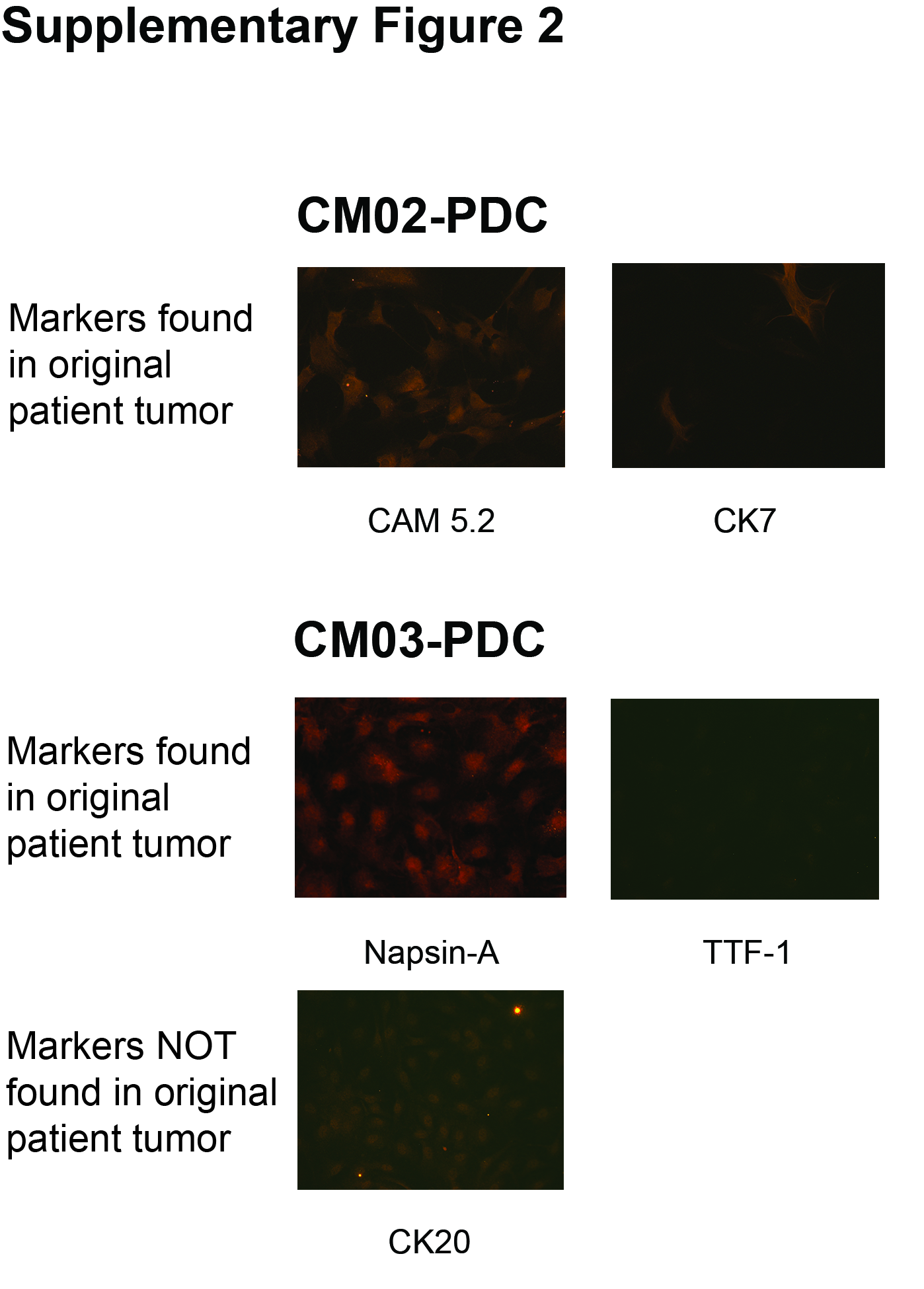

Supplement: Supplementary file 4 — Supplementary Figure 2 [file 41388_2019_680_MOESM4_ESM.tif]

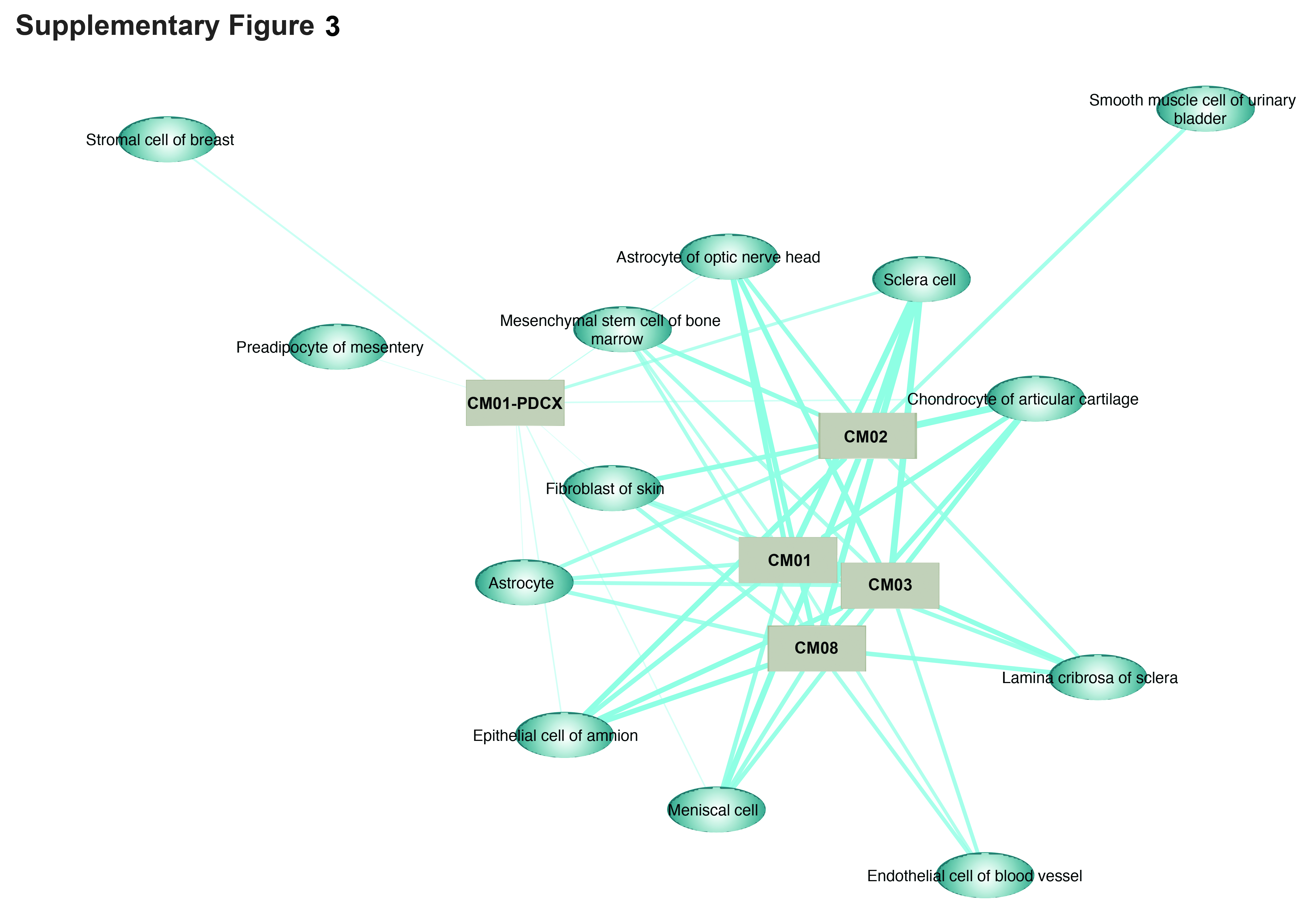

Supplement: Supplementary file 5 — Supplementary Figure 3 [file 41388_2019_680_MOESM5_ESM.tif]

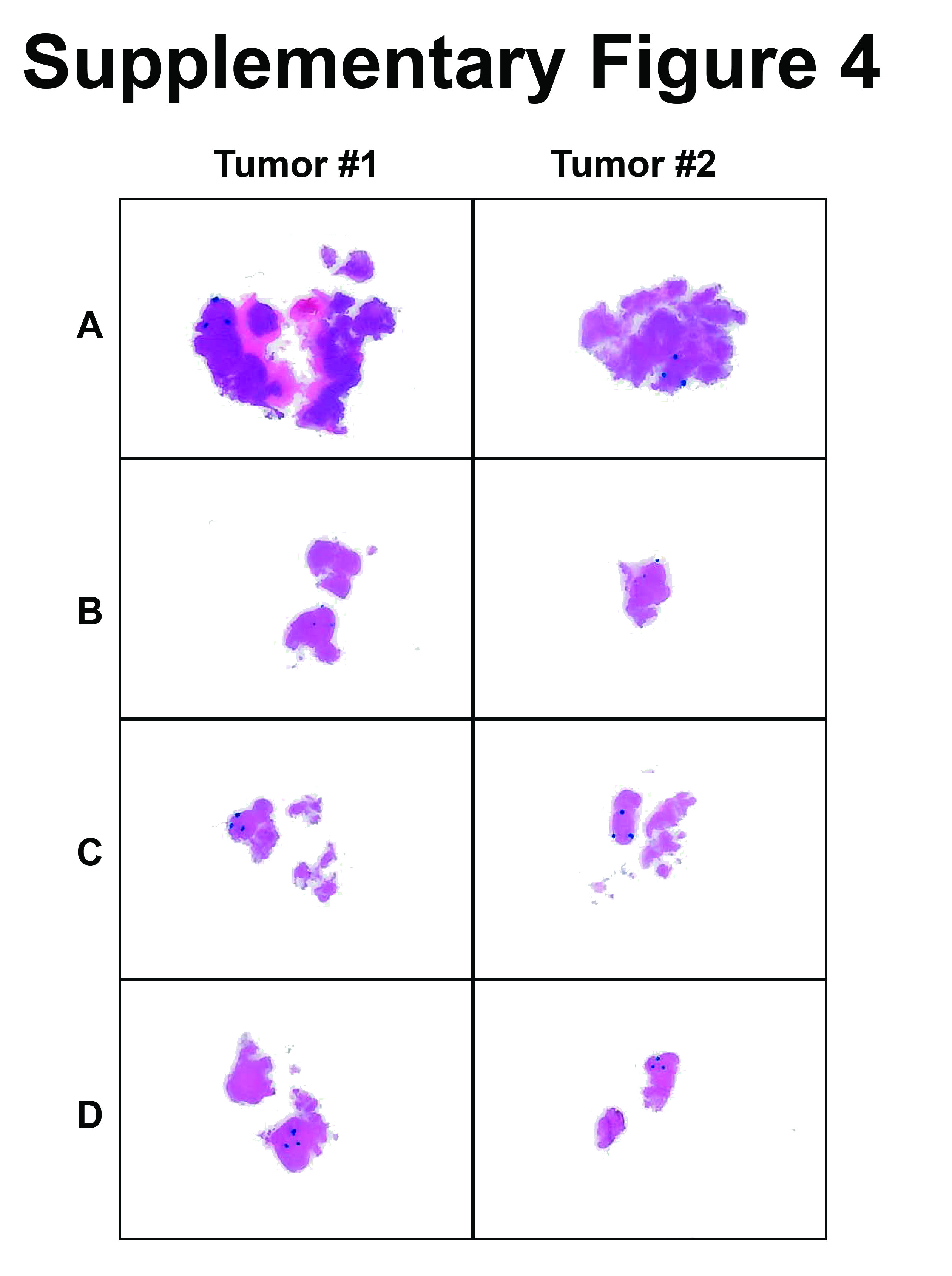

Supplement: Supplementary file 6 — Supplementary Figure 4 [file 41388_2019_680_MOESM6_ESM.tif]

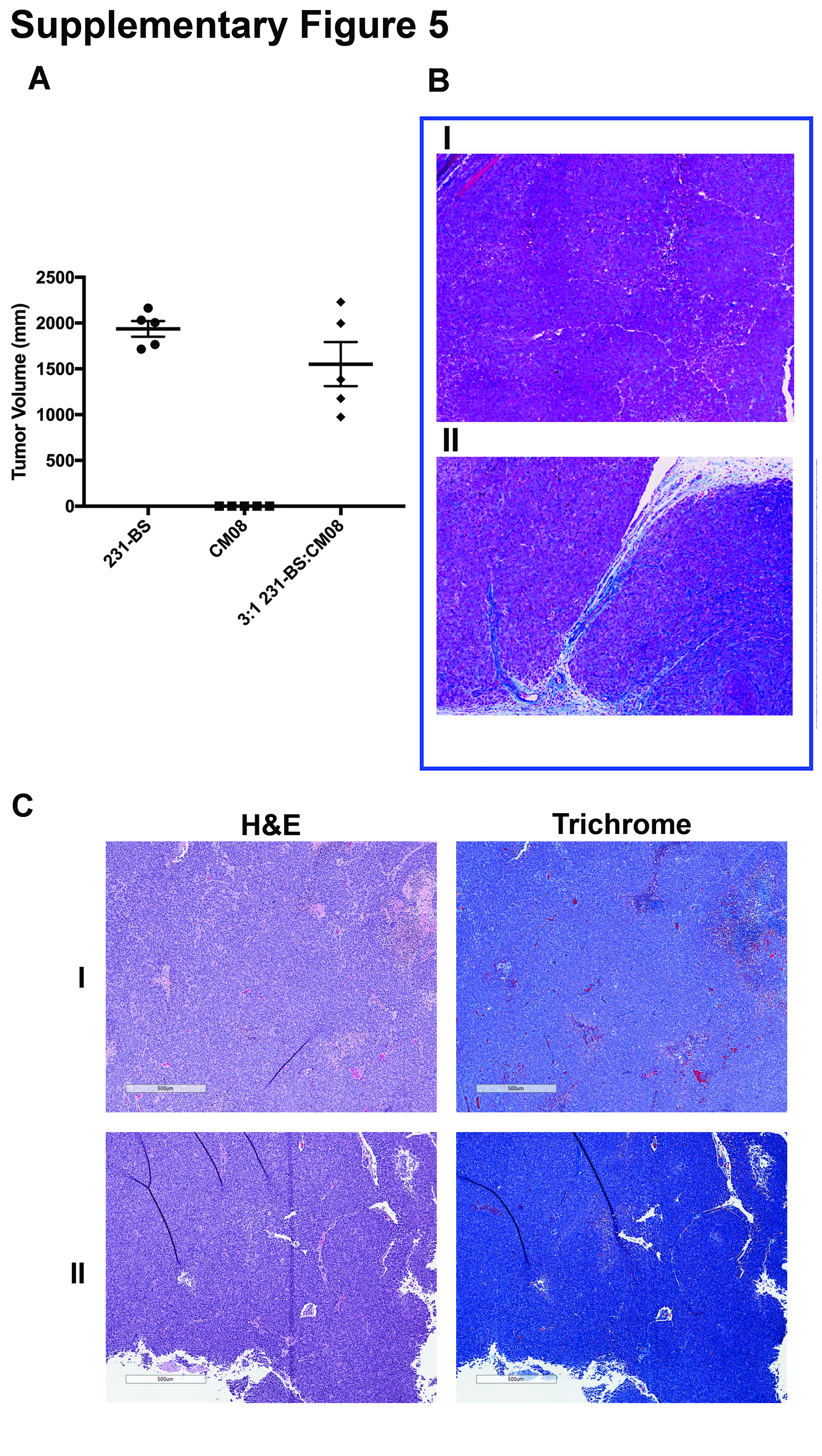

Supplement: Supplementary file 7 — Supplementary Figure 5 [file 41388_2019_680_MOESM7_ESM.tif]
